# 3D printed intelligent scaffold prevents recurrence and distal metastasis of breast cancer

**DOI:** 10.7150/thno.47933

**Published:** 2020-08-29

**Authors:** Xuelei Shi, Yanxiang Cheng, Jian Wang, Haoxiang Chen, Xiaocheng Wang, Xinghuan Li, Weihong Tan, Zhikai Tan

**Affiliations:** 1 College of Biology, Hunan University, Changsha, Hunan, 410082, China.; 2 Shenzhen Institute, Hunan University, Shenzhen, Guangdong, 518000, China.; 3 Department of Obstetrics and Gynecology, Renmin Hospital, Wuhan University, Wuhan, Hubei, 430060, China.

**Keywords:** Biofabrication, Environment response, Hemostasis, Prevention of tumor recurrence, Wound healing

## Abstract

**Rationale:** Tumors are commonly treated by resection, which usually leads to massive hemorrhage and tumor cell residues, thereby increasing the risk of local recurrence and distant metastasis.

**Methods:** Herein, an intelligent 3D-printed poly(lactic-co-glycolic acid), gelatin, and chitosan scaffold loaded with anti-cancer drugs was prepared that showed hemostatic function and good pH sensitivity.

**Results:** Following *in situ* implantation in wounds, the scaffolds absorbed hemorrhage and cell residues after surgery, and promoted wound healing. In an *in vivo* environment, the scaffold responded to the slightly acidic environment of the tumor to undergo sustained drug release to significantly inhibit the recurrence and growth of the tumor, and reduced drug toxicity, all without causing damage to healthy tissues and with good biocompatibility.

**Conclusions:** The multifunctional intelligent scaffold represents an excellent treatment modality for breast cancer following resection, and provides great potential for efficient cancer therapy.

## Introduction

Breast cancer is the most common malignant tumor among women worldwide. Approximately ~70-80% of patients who suffer from early non-metastatic disease will eventually be cured 1. Advanced breast cancer with distant organ metastases is considered incurable by the currently available treatment methods 2-4. The management of breast cancer is multidisciplinary, including both locoregional (surgery and radiation therapy) and systemic therapy approaches 3, 5. Conventional therapies, including systemic chemotherapy, are often faced with recurrence and metastases caused by residual cancer cells and circulating tumor cells (CTCs) 6, 7. Surgical tumor resection commonly results in significant hemorrhage and tumor cell residues, and microscopic lesions retained at the surgical resection edge and cells retained in the surgical wound are considered the main inducers of recurrence 8. In addition, intraoperative hemorrhage caused by damaged tissues and vasculature inevitably diffuses tumor cells into the circulation during tumor resection and increases the level of CTCs, thereby increasing the risk of local recurrence and distant metastases 9. In previous studies, it was shown that the unique environment of the lung is suitable for the residence and survival of cancer stem cells, making the lung a suitable place for the growth of metastatic cancer cells 10. Therefore, the development of a new local implant able to selectively kill residual tumor cells and simultaneously promote wound healing in postoperative treatment is of utmost importance.

To improve the efficacy of chemotherapy and reduce the possibility of recurrence and metastasis caused by residual cancer cells and CTCs, implants for local drug delivery have been developed 11, 12, including drug-loaded fibers 13, 14, films 15, 16, nanoparticles 17-20, and gels 21-23. Although these materials can exert the expected therapeutic effect, preventing cancer recurrence and metastases due to bleeding during surgery and poor wound closure are still major limitations. Therefore, an ideal multifunctional implant is needed to solve this issue. Compared with intravenous injection, nanoparticles 24, and liposomes 25, the implant proposed herein can reduce the loss of drug delivery. Furthermore, compared with films 22 and hydrogels, the implant is pH responsive, leading to controlled release that can reduce the risk of cancer metastasis and recurrence by local hemostasis and the absorption of free cells. In addition, the implant can accelerate the wound closure process, thereby reducing the poor prognosis caused by wound exposure. Biomaterials such as gelatin, chitosan, and poly(lactic-co-glycolic acid) (PLGA) have shown great potential as drug carriers 26. Gelatin, a hydrolysate of collagen with significant advantages in tissue engineering applications, is widely used in pharmaceutical and biomedical fields 27. Chitosan promotes wound healing through hemostasis and tissue regeneration 28. Gelatin and chitosan can be cross-linked to form Schiff base complexes, which have an imine bond (C = N-) that is pH responsive, resulting in easy hydrolysis under acidic conditions 29. PLGA is biodegradable and highly biocompatible 30, with its controlled degradation having been exploited to achieve the sustained release of drugs through wrapping of the required dose 31. The overall physical properties of a PLGA-drug matrix can be adjusted by controlling relevant parameters (e.g., polymer molecular weight and drug concentration). In addition, the required dose and release interval can be adjusted according to the drug type 32.

**Figure 1:**
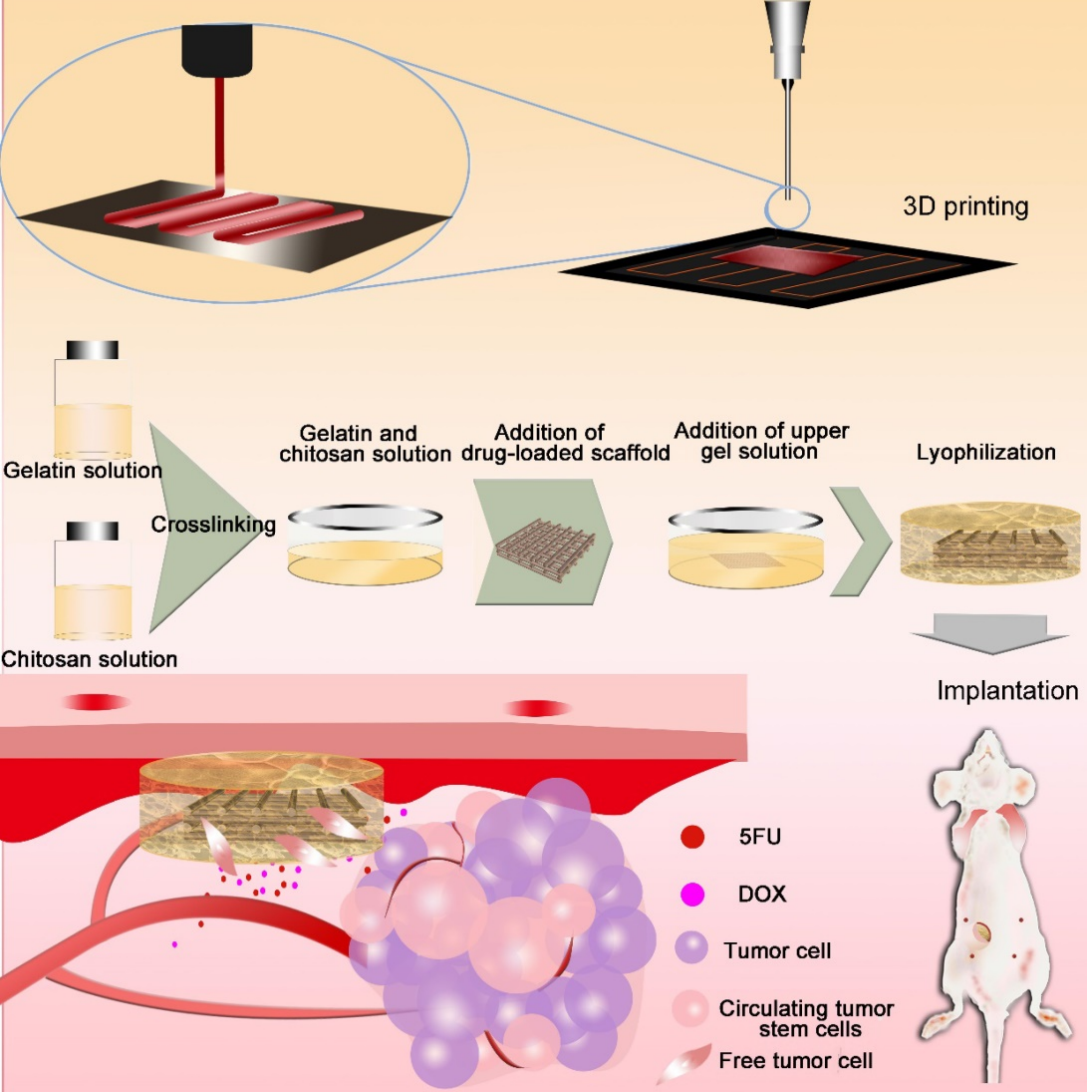
** Fabrication of the intelligent scaffold.** Drug-loaded scaffolds were printed by electro-hydrodynamic jet 3D printing, then sandwiched between a gelatin-chitosan gel. Scaffolds were implanted *in vivo* to absorb hemorrhage and cell residues after surgery, and to inhibit cancer cells and circulating tumor cells.

Three-dimensional (3D) printing 33-37, an additive manufacturing process that allows the manufacture of 3D entities of almost any shape, has been used to achieve personalized medicine 38, 39. 3D printing is simple and cost-effective, with extremely powerful functionalities in the development of drug delivery systems 40. Herein, we prepared drug-loaded scaffolds with a hemostatic function and good pH sensitivity using 3D printing. PLGA, gelatin, and chitosan were used to fabricate 3D scaffolds loaded with anti-cancer drugs (5-fluorouracil (5-FU) and doxorubicin hydrochloride (DOX)). The scaffolds had a sponge-like structure, could absorb blood, and inhibited cancer cell residues and CTC growth, thereby reducing the possibility of tumor recurrence. Responding to the tumor pH environment, the scaffold can intelligently control the release of loaded drugs and will eventually degrade *in vivo*. The multi-functional implanted scaffold can effectively prevent postoperative recurrence and distal metastases of tumors, thus providing significant potential for the integration of tumor therapy and wound healing after surgery (Figure 1).

## Methods

### Materials

5-FU was purchased from Sangon (Shanghai, China) and DOX was obtained from Meilun Biology (Dalian, China). PLGA in powder form (molecular weight of 5 × 10^5^ Dalton, with a lactide to glycolide ratio of 75:25) was bought from Daigang (Jinan, China) and gelatin was obtained from Dingguo (Beijing, China). Chitosan, 3-(4,5-dimethyl-2-thiazolyl)-2,5-diphenyl-2-H-tetrazolium bromide (MTT), and dimethyl sulfoxide (DMSO) were all obtained from Aladdin (Shanghai, China). Glutaraldehyde, N-N dimethylformamide (DMF), and citric acid were purchased from China Pharmaceutical Group (Beijing, China). Calcein (AM) and DAPI were purchased from Yesen Biotech (Shanghai, China). Hematoxylin and eosin (H&E) solution and the Masson dye kit were obtained from Soledad (Beijing, China).

### Preparation of intelligent drug-loaded scaffolds

5FU (20 mg) was dissolved into 3 mL of DMF, followed by the addition of DOX (9.9 mg) to obtain a DOX+5FU mixed solution; finally, PLGA (0.3 g) was added to the solution and stirred overnight to obtain a PLGA-DOX-5FU (PD5) solution. The solution was used for E-jet 3D printing to fabricate drug-loaded scaffolds (1 cm × 1 cm × 0.06 cm) according to our previous method 35.

Gelatin powder was dissolved in deionized water to obtain a 5% gelatin solution and chitosan was dissolved in 10% citric acid solution to obtain a 2.5% chitosan solution. Next, gelatin and chitosan were added at a volume ratio of 2:1 to form a mixed solution, and 20 μL of glutaraldehyde was added per milliliter of mixed solution to achieve crosslinking at 37 ºC for 30 min. Subsequently, the PD5 scaffold was sandwiched between the gelatin-chitosan (GC) gel, and dried using a freeze dryer (Free Zone, Labconco, USA). Subsequently, the intelligent scaffold (IS) was collected (Figure 1).

### Characterization of functional scaffolds

The PD5 scaffold was analyzed using an ultraviolet spectrophotometer (NanoDrop2000, Thermofly, USA). Fabricated scaffolds were then scanned with a micro-CT (skyscan-1276, Bruker, Belgium) under the following scanning conditions: exposure (ms) = 360, voltage (kV) = 43, current (µA) = 200. A field emission scanning electron microscope (JSM-6700F, Japan) and an environmental scanning electron microscope (FEI QuANTA 200, Czech Republic) were used for surface characterization. An energy dispersive spectrometer (EDS, JSM-6700F, Japan) was employed to analyze the components.

### Biosafety assessment

MDA-MB-231, NIH3T3 and HUVEC cells were inoculated into culture dishes with IS respectively, and untreated cells served as the control group. The cell viability was obtained by the MTT method, and the survival and proliferation ability of cells were quantified by immunofluorescence staining.

### Blood absorption capacity of scaffolds

Blood (10 µL) from Kunming mice was placed into culture dishes, dried GC scaffolds, and the IS. After incubation at 37 °C for 5 min, the same amount of deionized water was added, and of each sample, the absorbance at 540 nm was measured using an ultraviolet spectrophotometer (NanoDrop2000, Thermofly, USA). Dried GC and IS with the same weight were placed into deionized water or blood, retrieved, the excess liquid was removed, and finally weighed.

The absorptivity was calculated using the following formula:

Absorptivity (%) = (m-m_0_)/m_0_ × 100%;

where m is the weight of soaked GC or IS, and m_0_ is the weight of dried GC or IS.

To quantify the clotting ability of the material, the blood clotting index (BCI) was calculated. The higher the BCI value, the worse the blood coagulation ability of a material. Assuming that the absorbance value of whole blood in deionized water at 540 nm is 100 (as a reference), the BCI of the material was calculated by the following formula:

BCI = absorbance of blood after contact with material at 540 nm/absorbance of whole blood in deionized water at 540 nm × 100

### Wound healing assessment

For the wound healing assessment, Kunming mice were used. All animal experiments were approved by the Animal Experimental Ethics Committee of Hunan Experimental Animal Center (license number: SYXK (Xiang) 2013-0001). Mice were divided into three groups before surgery and anesthetized by intraperitoneal injection of pentobarbital sodium (Sigma, Missouri, USA). An iodine solution was used to disinfect the shaved part of the mouse abdomen and a round full-thickness skin wound (about 10 mm) was created near the breast part of the right abdomen. Next, one group of mice that were treated with gauze served as the control group and the other two groups were treated with PD5 and IS scaffolds, respectively. The wound area was observed and measured weekly. Mice were sacrificed at the designated time (5, 10, and 30 days after implantation), and corresponding skin tissues were collected for H&E and Masson staining.

### *In vitro* anti-tumor activity

#### Drug release efficiency

To measure the drug release, the IS was immersed into 20 mL of aqueous solutions at 37 °C, at pH 5.0, 6.5, and 7.0, respectively. A 3-μL aliquot of the solution was taken every day and measured at 250 and 265 nm using an ultraviolet spectrophotometer (NanoDrop2000, Thermofly, USA) to obtain the concentrations of released 5FU and DOX (concentration = absorption coefficient × absorbance; while the absorption coefficient of 5FU is 114.85 and the absorption coefficient of DOX is 3.18).

#### Synergy of drugs

Human triple-negative breast cancer cells (cell line MDA-MB-231) was provided by Prof. Yongjun Tan (Hunan University, China). The cells growing in the logarithmic phase were inoculated into 96-well plates (1 × 10^5^ cells/well). Following cell adhesion for 12 h, the different drug concentrations were respectively added. After 24 h, 40 μL of the MTT solution were added in the dark and incubated at 37 °C for 4 h. Subsequently, culture supernatants were removed and 150 μL of the MTT solution were added to each well and shaken at low speed for 15 min. After that, the absorbance of each well was detected at 490 nm with a microplate reader (EnSpire 2300, PerkinElmer, Singapore), and the synergistic effect of the two different drugs (SI) was evaluated by the following formula 41:

SI = D_(DOX)_/D_x(DOX)_ + D_(5FU)_/D_x(5FU)_

where D_x(DOX)_ and D_x(5FU)_ represent the inhibitory concentrations (IC_X_) of DOX and 5FU, respectively, and D_(DOX)_ and D_(5FU)_ represent the concentrations of DOX and 5FU in the IC_X_ value in the mixed dual drugs, where SI > 1 represents drug antagonism, SI = 1 represents addition, and SI < 1 represents synergy.

#### Cytotoxicity assay

MDA-MB-231 cells were cultured in 10% FBS and 1% antibiotics medium (5 mL) in a 10-cm culture dish (3 × 10^5^ cells/dish) for 12 h. Next, DOX (0.94 μM) and 5FU (0.31 mM) mixed solutions, the drug-loaded PD5 scaffolds, and the IS were added to the respective wells. Untreated cells were used as the control. After 24 and 48 h, the samples were collected and subjected to further testing. Fibroblasts (NIH3T3) and human vascular endothelial cells (HUVEC; both obtained from the Type Culture Collection of the Chinese Academy of Sciences, Shanghai, China) were also cultured on IS, and used as the control.

Calcein (0.25 μL/mL) was used for live/dead staining. For immunofluorescence staining, cells were fixed with paraformaldehyde (4%) for 10 min, wells were rinsed with phosphate buffer saline (PBS), and 0.4% Triton-100 was used to permeabilize the cells. Next, cells were blocked using sheep serum (Cat No. AR1009, Boster, Wuhan, China) for 30 min, and incubated with primary antibodies at 4 ºC (Ki67, Cat No. 27309-1-AP; Bcl-2, Cat No. 12789-1-AP; Bax, Cat No. 50599-2-Ig; all from Proteintech, Wuhan, China, dilution ratio is 1:100). The cells were then incubated with a secondary antibody for 3 h at room temperature (Cat No. 33107ES60, Yeasen, Shanghai, China), rinsed with PBS, and incubated with DAPI for 15 min. All samples were observed using an Olympus confocal laser scanning microscope (FV1000, Japan).

### *In vivo* anti-tumor activity

MDA-MB-231 cells were injected into the inguinal (4th from top) right mammary fat pad of 24 female BALB/C nude mice (4 × 10^6^ cells/mouse) to establish orthotopic breast tumors. Mice were divided into four groups: the control group (no treatment); the dual-drug group (intravenously injected with DOX (1 mg/kg) and 5FU (6 mg/kg) mixed solution every 3 days); the PD5 group (implanted with PD5 drug-loaded scaffold), and the IS group (implanted with the IS). When the tumor volume reached 200 mm^3^, the tumor was surgically removed, and tumor tissue with a diameter of ~2 mm was retained during the resection. Postoperative observation was performed every 3 days. The weight of mice and the volume of any recurrent tumors were assessed. After 1 month, mice were scanned using nuclear magnetic resonance imaging to assess the recurrence of tumors. The corresponding recurrence rate was obtained by dividing the number of mice with tumor recurrence by the total number of mice per group. Relevant tissues and tumors were obtained after sacrificing mice for further histopathological analysis.

### Histopathological analysis

Tissues obtained were fixed with 4% paraformaldehyde for 24 h, rinsed with PBS, then rinsed with 70% alcohol. The samples were then incubated overnight in 70% alcohol, dehydrated with different concentrations of alcohol, and treated with xylene. Finally, the samples were embedded in paraffin for 2 h and paraffin sections were prepared. Sections were stained with H&E and Masson, and observed using a microscope.

### Statistical analysis

Data were obtained from at least five replicates and expressed as the mean ± standard deviation. One-way ANOVA with a Tukey post hoc test was performed to determine the statistical significance, and* P* < 0.05 was considered statistically significant.

## Results

### Fabrication of functional scaffolds

**Figure 2:**
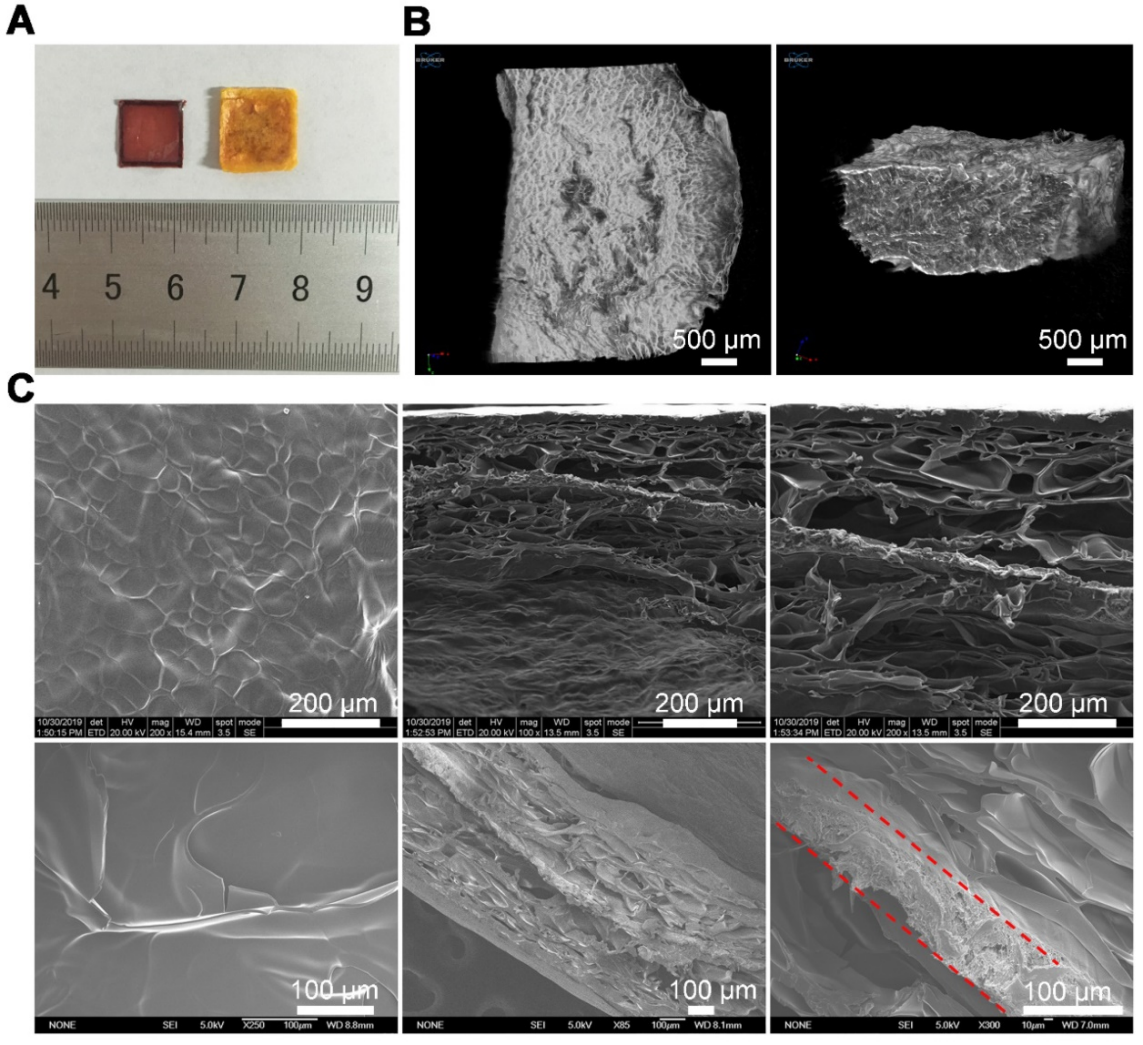
** Preparation and characterization of the intelligent scaffold. (A)** Optical images of the PLGA-DOX-5FU (PD5) scaffold (left) and the intelligent scaffold (IS, right). **(B)** Micro-CT images of the IS. **(C)** Images of the IS using a field emission scanning electron microscope (upper row) and an environmental scanning electron microscope (bottom row, the dotted lines indicate the PD5 scaffold).

The drug-loaded PLGA-DOX-5FU (PD5) scaffolds were fabricated using our electro-hydrodynamic jet (E-jet) 3D printing system 42, 43 and characterized by micro-CT and scanning electron microscopy (SEM; Figure S1, Figure S2). To ensure pH responsiveness, the PD5 scaffolds were sandwiched between a GC gel to fabricate the intelligent scaffold (IS) (Figure 1), which were shown to be completely wrapped by the gel (Figure 2A, Figure S3, and Figure S4). The micro-CT and fluorescence images showed that the outer layer of the IS had a porous sponge-like structure (Figure 2B, Figure S5), as verified by SEM. Compared with the GC scaffold, the IS had better mechanical properties (Figure S6). Furthermore, the scaffolds had a layered structure, proving that the PD5 scaffold was sandwiched between two GC layers (Figure 2C).

### Biofunctionality of the IS

**Figure 3:**
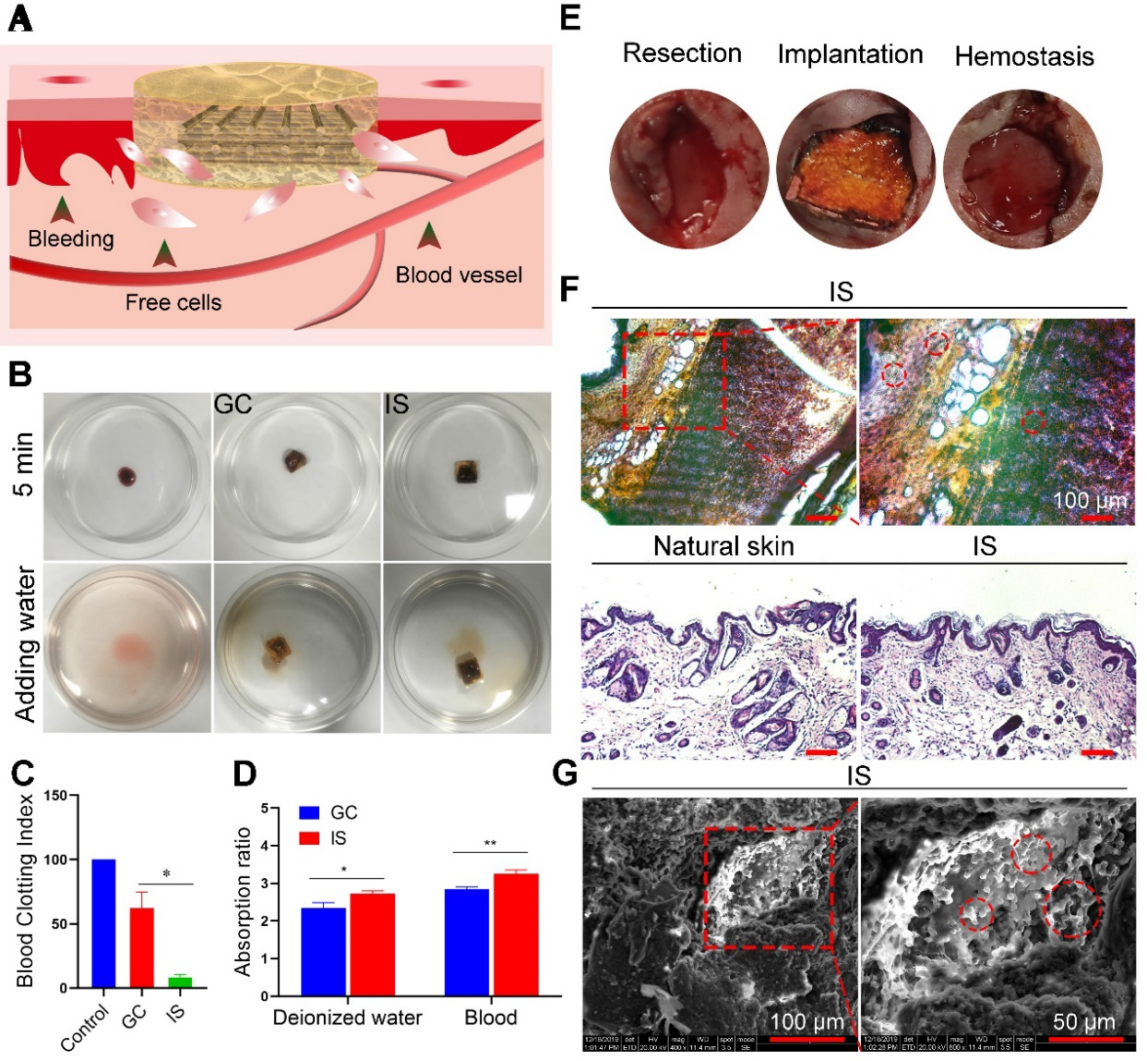
** Biocompatibility and cell viability of the intelligent scaffold. (A)** Schematic diagram of using the intelligent scaffold (IS) to a bleeding site. **(B)** Coagulation ability of the gelatin-chitosan (GC) scaffold and IS *in vitro*. **(C)** Blood clotting index of GC scaffold and IS. **(D)** Water and blood absorption ability of GC scaffold and IS. **(E)** Hemostasis of IS* in vivo*. **(F)** H&E staining of IS (upper row) and skins (bottom row: natural skin tissue and regenerated tissue near the IS) after implantation for 30 days. **(G)** SEM images of IS after implantation for 30 days (red circles indicate absorbed and infiltrated cells). *P < 0.05, **P < 0.01.

To explore the biocompatibility and hemostasis ability of the IS, both *in vitro* and *in vivo* experiments were performed (Figure 3A). For *in vitro* assessment, fresh blood was placed into culture dishes (control), on GC scaffolds, and the IS, incubated at 37 °C for 5 min, then deionized water was added (Figure 3B). A higher BCI value indicates a lower blood clotting ability 44. The absorbance of different treatment groups at 540 nm was measured to obtain the corresponding coagulation index (Figure 3C) and absorption capacity (Figure 3D). H&E and fluorescence staining analysis also showed that the scaffold absorbed free cells in blood (Figure S7 and Figure S8). As expected, the BCI of the GC scaffolds (62) was 7.5-fold higher than that of the IS (7.42), demonstrating the good clotting ability of IS. The blood absorption rate of the IS group (3.3) was 1.2-fold higher compared to that of the GC group (2.7). The results show that the deionized water and blood absorption capacity of IS was greater than that of the GC scaffold, likely due to the higher porosity of IS. To assess the biocompatibility of the scaffolds, MDA-MB-231, NIH3T3, and HUVEC cells were cultured with IS. Cells were stained on days 1 and 2 after incubation, and the number of cells was quantitatively analyzed. The results showed that all the cells had good adhesion and proliferation ability on the scaffolds. In addition, the cell viability was measured by MTT assay. The results demonstrated that the IS had good biocompatibility, with no obvious adverse effects on cell viability after 2 days of treatment (Figure S9-S12). For *in vivo* assessment, the IS was applied to the wound of mice and its hemostatic ability was observed after 5 min. The results showed that IS can stop bleeding by absorbing exuded blood, solidifying the blood, and compressing the bleeding site (Figure 3E). Furthermore, the IS was implanted into mice for 30 days, and the cell absorption ability was evaluated by staining the scaffold sections and skin (natural skin tissue and regenerated tissue near the IS, Figure 3F). H&E staining and SEM of the IS showed that many cells resided in the scaffold, thereby indicating that the scaffold was viable for cells (Figure 3G). The results showed that the IS had good hemostasis and cell viability.

### Wound healing assessment

**Figure 4:**
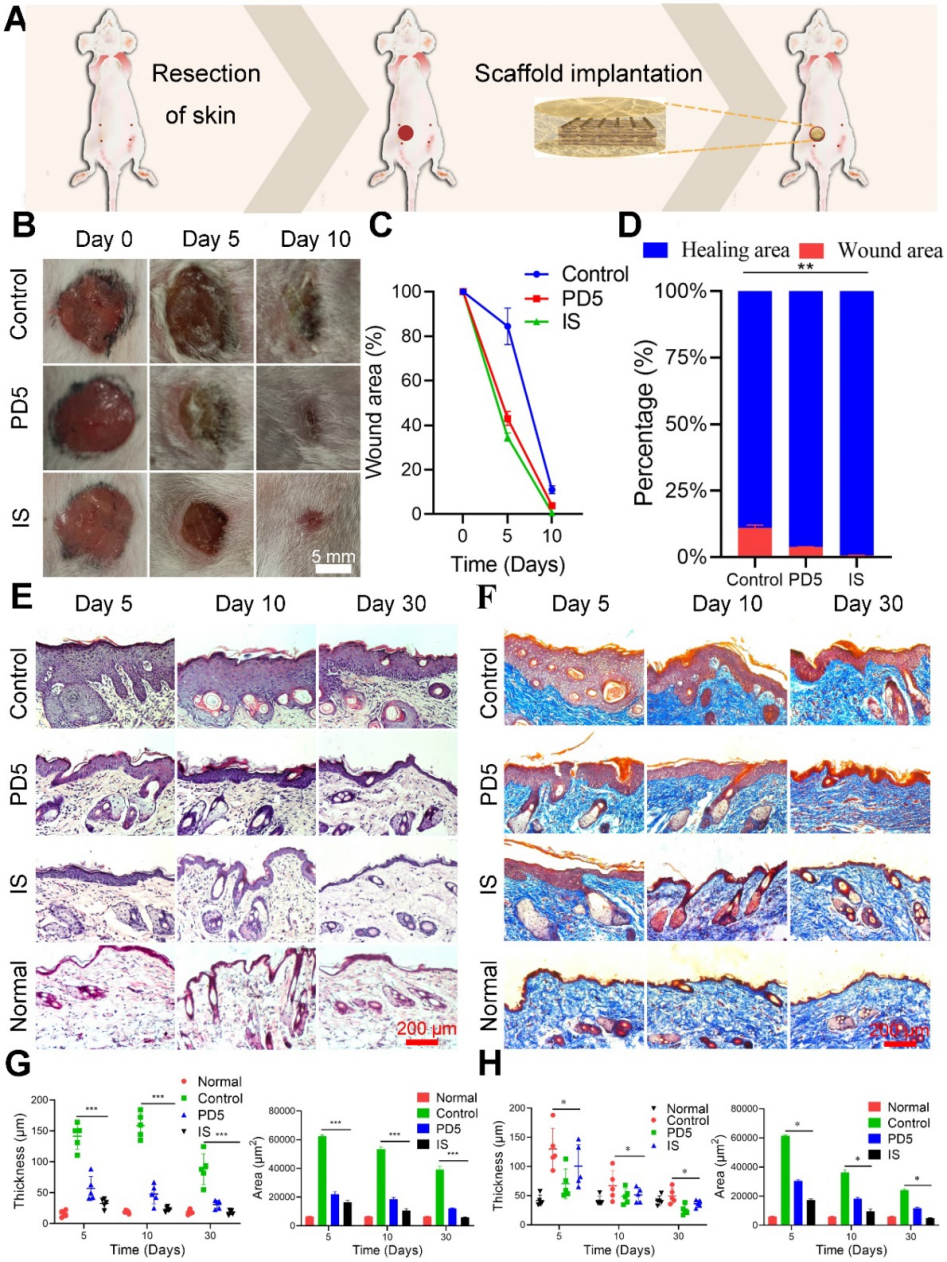
** Skin repair assessment using the various scaffolds. (A)** Schematic diagram of the process. **(B)** Images of wound healing of mice treated with gauze (control), PD5 scaffold, and the intelligent scaffold (IS). **(C)** Quantification of wound closure within 10 days. **(D)** The ratios of wound closure and residual area after 30 days. **(E)** H&E staining images of normal skin and wound area after treatment for 5, 10, and 30 days with control, PD5 scaffold, and IS. **(F)** Masson staining images of normal skin and wound area after treatment for 5, 10, and 30 days with control, PD5 scaffold, and IS. **(G)** Quantification of the epidermal thickness and area of different groups from the H&E staining images. **(H)** Quantification of the epidermal thickness and area of different groups from the Masson staining images. *P < 0.05, **P < 0.01, ***P < 0.005.

To evaluate the skin repair ability of the scaffold, a full thickness skin defect model was established near the breast of mice, and the wound was covered with the scaffolds (Figure 4A). Therapeutic effects were evaluated on days 0, 5, and 10 (Figure 4B). On day 5, the wound area of the control group (treated with gauze) was 84%, while that of the PD5 and IS groups were 43% and 34%, respectively. Thus both the PD5 and IS scaffolds had the ability to promote wound healing, with IS achieving the best results (Figure 4C and Figure 4D). To explore the skin repair efficacy of different treatment groups, H&E (Figure 4E) and Masson staining (Figure 4F) were performed on skin tissue from the wound area of the different treatment groups on days 5, 10, and 30 for histological morphology. Fibroblasts play a critical role in normal wound healing 45. Furthermore, the outer hydrogel of the IS can absorb and retain wound exudate, and promote the proliferation of fibroblasts and consequently the formation of wound epithelium 46, 47. Compared with the control and PD5 groups, the amount of deposited collagen in the IS group initially increased and then decreased. In the early stage of wound healing, an increase in collagen is beneficial to wound healing, yet excessive collagen will subsequently lead to scar tissue formation 48. The epidermal thickness and area of the different groups were quantitatively compared (Figure 4G and Figure 4H), showing that, after 30 days of treatment, the IS group had the least epidermis thickness and area. Additionally, regenerated skin tissue in the IS group exhibited the most similar tissue structure to healthy skin tissue, thereby demonstrating that the IS resulted in efficient skin repair.

### *In vitro* anti-tumor activity

**Figure 5:**
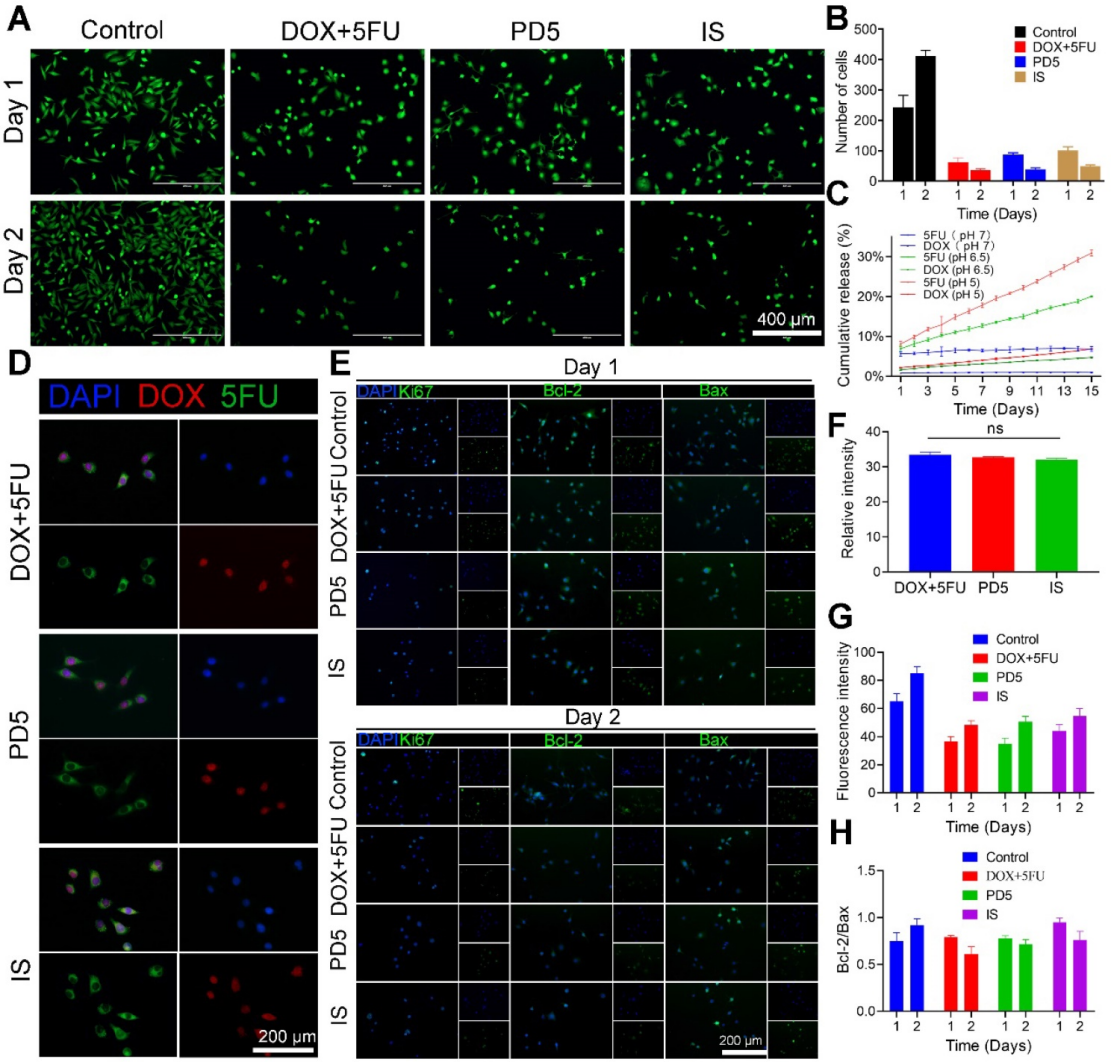
***In vitro* anti-tumor activity. (A)** Live cell staining of cells treated with DOX+5FU mixed solutions, the PD5 scaffold, and the intelligent scaffold (IS), respectively. Untreated cells were used as the control. **(B)** The number of surviving cells after the respective treatments. **(C)** Drug release profile of the IS under different pH values. **(D)** The drug uptake in cells after treatment for 10 h. **(E)** Immunofluorescence staining of treated cells on days 1 and 2. **(F)** Quantification of DOX fluorescence intensity of cells in panel D. **(G)** Quantification of Ki67 fluorescence intensity in cells on days 1 and 2 from panel E. **(H)** Quantification of the Bcl-2/Bax ratio on days 1 and 2 from panel E.

To investigate the anti-tumor efficacy of IS, MDA-MB-231 cells were treated with DOX (0.94μM) and 5FU (0.31mM) mixed solutions (DOX+5FU), PD5, and the IS, respectively. Untreated cells served as the control (Figure 5A and Figure S13). On the first day, the average cell number in the DOX+5FU group was about a quarter of that in the control group. The DOX+5FU dual-drug group showed stronger cell growth inhibition. However, on the second day, the cell growth inhibition was similar for both the dual-drug group and the IS group (Figure 5B), which was likely due to the scaffold's response to the acidic tumor environment, resulting in a greater release of drugs. Thus, IS has a good inhibitory effect on tumor cell proliferation. Furthermore, the drug release profile of IS under changing pH was assessed, and the results showed that the IS responded to the acidic tumor environment, thus accelerating drug release (Figure 5C).

The combined use of 5FU and DOX has a synergistic effect (Figure S14). To assess cellular drug uptake following drug release from the different scaffolds, cells that underwent the various treatments (DOX+5FU, PD5, and the IS) were observed using a confocal microscope 10 h later (Figure 5D and Figure S15). The fluorescence intensity of drug uptake in cells from different treatment groups was quantified (Figure 5F). No significant differences were observed at 10 h. Furthermore, Ki67, Bcl-2, Bax, and DAPI staining were also performed on all cells (Figure 5E) and then their fluorescence was quantified to study the expression of different proteins in cells (Figure 5G). The mean fluorescence intensities of the cells in the IS group on days 1 and 2 were 65.07 and 85.03, respectively, while those in the control group were 44.21 and 54.66, respectively. The results showed that, compared with the control group, the IS group showed lower Ki67 expression. Moreover, the ratio of Bcl-2/Bax on days 1 and 2 was 0.75 and 0.92 in the control group, 0.79 and 0.60 in the DOX+5FU group, 0.78 and 0.69 in the GC group, and 0.95 and 0.76 in the IS group, respectively (Figure 5H). The Bcl-2/Bax ratio increased in the control group on the second day, and decreased in the other three groups, thereby indicating that the IS had a significant cytotoxic effect on tumor cells.

### Therapeutic efficacy

**Figure 6:**
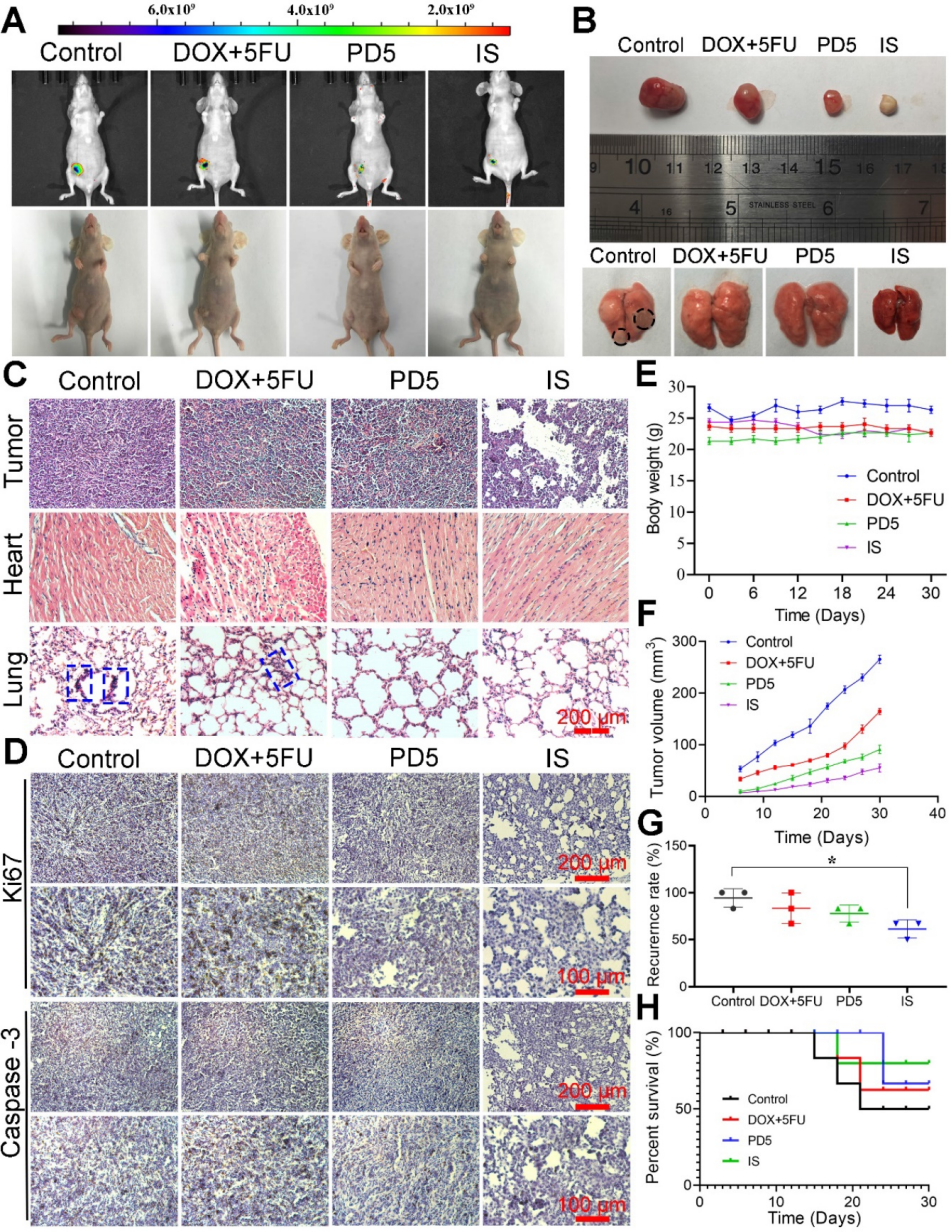
** Therapeutic efficacy of scaffolds. (A)** Visible light images and *in vivo* fluorescence images of nude mice after 30 days of treatment. **(B)** Photographs of recurrent tumors and lungs after 30 days of treatment. **(C)** H&E staining of recurrent tumors, hearts, and lungs (the blue dotted frames indicate the tumor lesions). **(D)** Immunohistochemical analysis of sections of recurrent tumor stained with anti-Caspase-3 and anti-Ki67 antibodies. **(E)** Changes in body weight and **(F)** growth of recurrent tumor during various treatments. **(G)** Postoperative recurrence rate and **(H)** survival curve of mice during various treatments. *P < 0.05.

The *in vivo* anti-tumor ability of the IS was observed for up to 30 days. Compared with other groups, the IS group showed the lowest increase in tumor growth, thus indicating its good anti-tumor efficacy (Figure 6A). Tumors, corresponding tissues, and organs were obtained after sacrificing mice; the tumor size and lung metastases conditions of the different groups (Figure 6B) as well as their histological changes (Figure 6C) were observed. Pathological changes (tumor lesions) were observed in the lungs of mice in the control group, however, no significant changes were observed in other groups. Lung metastases were observed in the control and the DOX+5FU groups, but not in the IS and PD5 groups. Furthermore when compared with the PD5 group, the IS group had the best therapeutic effect, indicating that the drug-loaded scaffolds not only inhibited tumor growth, but also reduced the risk of distal metastasis. Mice tumors were immunohistochemically stained for Ki67 and Caspase-3 to explore their expression in tumors (Figure 6D). The results showed that, compared with PD5 and IS groups, Ki67 expression was the highest in the control and the DOX+5FU groups, and Caspase-3 expression was not significantly different between groups, indicating that the drug-loaded scaffolds can inhibit cell proliferation and thus restrain tumor growth. To evaluate the effects of the different treatments on mice as well as on the therapeutic effects on tumors, changes in body weight and tumor volume were monitored during the treatment (Figure 6E-F) and the tumor recurrence and survival rates of mice were recorded (Figure 6G-H). The average tumor volume in the control group was 265.47 mm^3^, while that in the DOX+5FU, GC, and IS groups was 164.39 mm^3^, 90.93 mm^3^, and 55.07 mm^3^, respectively. The survival rate in the IS group was 80%, while those in the control, DOX+5FU, and GC groups were 50%, 62.5%, and 66.7%, respectively. The recurrence rate of the IS group was 61.33%, which was the lowest of all groups. During treatment, no significant changes in body weight were observed. However, the tumor growth rate of the IS group was slower than that of the other groups, with a lower tumor recurrence rate and a higher survival rate than in the other groups. Liver, spleen, and kidney tissues of mice were sectioned and observed, and no damage was observed (Figure S16).

## Discussion

Herein, we used the E-jet 3D printing system to fabricate an IS that was loaded with the drugs DOX and 5FU. The micro-CT and SEM images showed that the drug-loaded scaffolds were wrapped with the pH-responsive GC gel, with a sponge-like structure. The sandwich-like structure provided the IS with the ability to cause hemostasis and promote cell growth and attachment. Gelatin can activate platelet aggregation and can be used as an absorbable hemostatic agent 49. Chitosan has an excellent hemostasis performance as it can form cation clusters and interact with anions on red blood cells, thus inducing platelet aggregation and eventually preventing blood loss. In addition, chitosan has a good film-forming ability and high viscosity, with the ability to coordinate and crosslink with gelatin 50. The above properties enable gelatin and chitosan to form natural semi-interpenetrating polymer networks forming biomaterials with a porous structure similar to the biological extracellular matrix 51. The grid scaffold manufactured by 3D printing has a high specific surface area, which can accelerate the release of drugs following responding to the pH. Due to the special porous structure of the scaffold, blood can rapidly be absorbed and coagulated. Therefore, the IS not only reduces the loss of drug delivery but can also respond to the acidic environment of the tumor, thereby leading to a sufficient anti-tumor effect. Furthermore, it can reduce the recurrence and distant metastasis of tumors, and improve the survival rate of patients through local hemostasis and the uptake of free cancer cells. The *in vivo* and *in vitro* results showed that the IS has good hemostasis ability. Furthermore, the IS was shown to be able to absorb extravasated blood and free tumor cells released by surgery. Tissue sections of normal organs (liver, spleen, and kidney) from mice treated with IS showed no obvious toxic effects, indicating that the scaffold had good biocompatibility.

To evaluate the ability of the IS to promote wound healing, a full-thickness skin defect model was established near the breast of mice. The experimental group experienced a greater healing effect than the control. H&E and Masson staining showed that, compared with the control, the healing site of the experimental group was similar to that in healthy skin, with comparable skin thickness and structure. The IS reduced the formation of scar tissue, likely due to the decrease in collagen. The presence of collagen is beneficial for wound repair at the early stage of wound healing, however, a large amount of collagen secretion leads to scar formation 48. Taken together, these results indicated that the IS has a good wound healing ability.

The pH dependent drug-release ability of the IS was evaluated by treating the scaffold under different pH environments. The release efficiency was greater in an acidic environment similar to that of the tumor microenvironment 52. The IS showed significant cytotoxicity on tumor cells after 2 days of treatment, as evaluated by live/dead staining. This was attributed to drug release in response to the acidic tumor environment. The drug-uptake ability of tumor cells was assessed after 10 h of treatment. Cells in the IS group showed a similar uptake ability to those in the DOX-5FU group, showing that drug-loaded scaffolds have a high drug delivery efficiency and are able to target cells.

The Bcl-2 family is composed of various pro-apoptotic and anti-apoptotic proteins, the interaction of which regulates the key balance between cell life and death 53. Bax is a main cytoplasmic protein and its activation and translocation is required to trigger mitochondria to induce mitochondrial outer membrane permeability 54. If the ratio of Bcl-2 to Bax decreases, Bax triggers the release of cytochrome C, which eventually leads to cell apoptosis 55. Ki67, Bcl-2, and Bax staining in tumor cells revealed that, by increasing the incubation time, both the expression of Ki67 and the ratio of Bcl-2/Bax decreases in the IS group when compared with the control group, indicating that the IS has a good killing ability on tumor cells.

Bleeding after tumor resection may lead to the diffusion of residual tumor cells, thereby increasing the number of CTCs in blood, leading to an increase in the risk of tumor recurrence 56. To evaluate the efficacy of the IS to reduce the risk of recurrence and distal metastasis after operation, the IS was implanted into nude mice following tumor resection. The cross-linking of chitosan and gelatin in the IS leads to the formation of Schiff base complexes, which respond to the acidic environment of recurrent tumors, thereby releasing the loaded drugs. The size of the recurrent tumor and other tissues were observed after 30 days of implantation, with the IS having a great anti-tumor effect and no toxic effects on the body. Therefore, scaffolds can degrade slowly *in vivo* and have no side effects (Figure S15 and Figure S17). Compared with the control group, the IS group showed no obvious pathological changes or metastasis.

Adriamycin (DOX) is a widely used anticancer drug that induces dose-related cardiotoxicity 57. H&E staining of the tumor, heart, and lung tissues revealed that the IS not only had a good tumor inhibition effect but also was able to reduce the DOX toxicity to the heart when compared with the dual drug group. Compared with other groups, mice treated with the IS exhibited less weight change, a higher survival rate, and a lower recurrence rate. Immunohistochemical analysis of Ki67 and Caspase-3 showed that the expression of Caspase-3 was not high in the IS group, and that the relative expression of Ki67 was also lower, likely due to the slow release effect of the IS inhibiting the growth of recurrent tumors. The IS can absorb blood and exfoliated tumor cells, thus reducing the possibility of recurrence. Furthermore, the IS reduced drug toxicity with a low rate of postoperative anti-tumor recurrence and metastases.

## Conclusion

Herein, we prepared an IS with a good blood absorption and cell residence ability, pH response that could promote rapid wound healing. Therefore, the IS has significant potential in reducing tumor recurrence after breast cancer surgery. In an *in vivo* environment, the scaffold can respond to the slightly acidic tumor environment to undergo sustained drug release, thereby significantly inhibiting the recurrence and growth of the tumor and reducing the drug toxicity, all without causing damage to normal tissues and with good biocompatibility. Thus, the multifunctional IS is a promising treatment choice for breast cancer following resection, with great potential for efficient cancer therapy.
