# Supplementary material for: 3D printed intelligent scaffold prevents recurrence and distal metastasis of breast cancer
Source: Theranostics. 2020 Aug 29;10(23):10652–64. doi: 10.7150/thno.47933 (PMC7482818; doi:10.7150/thno.47933)
Supplement: Supplementary file 1 — Supplementary figures. [file thnov10p10652s1.pdf]

Supporting information

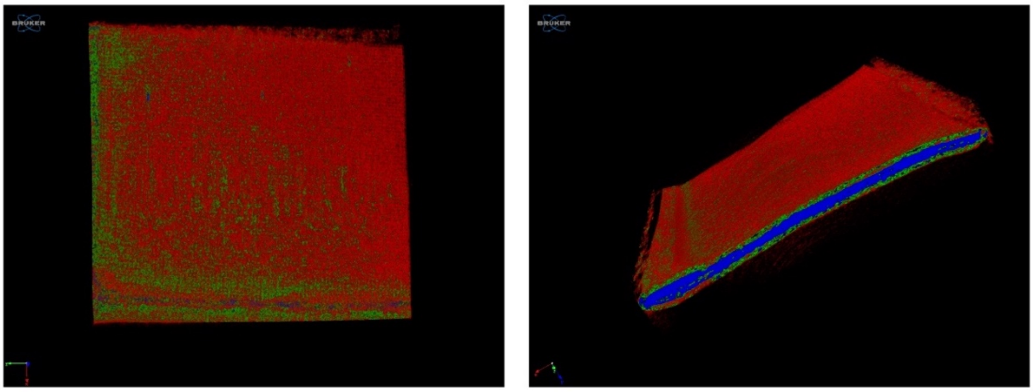

Figure S1. Micro-CT images of the PLGA-DOX-5FU scaffold.

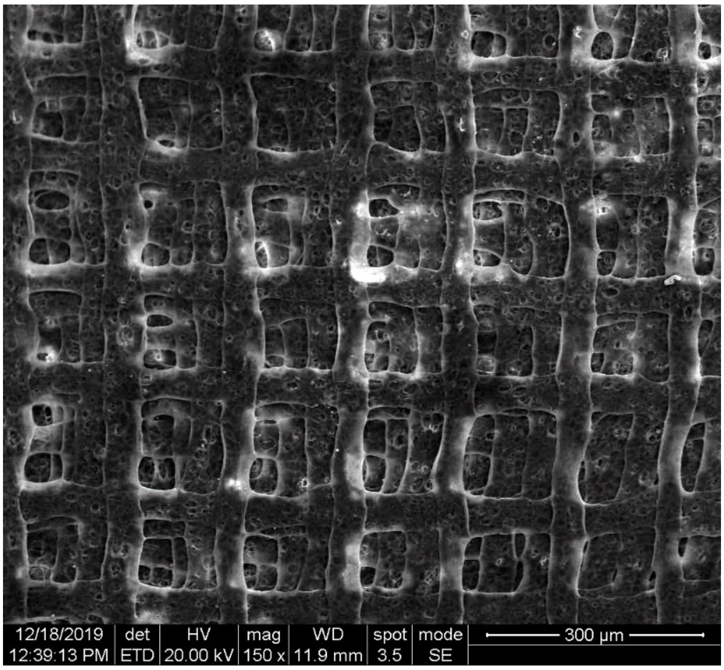

Figure S2. SEM images of the PLGA-DOX-5FU scaffold.

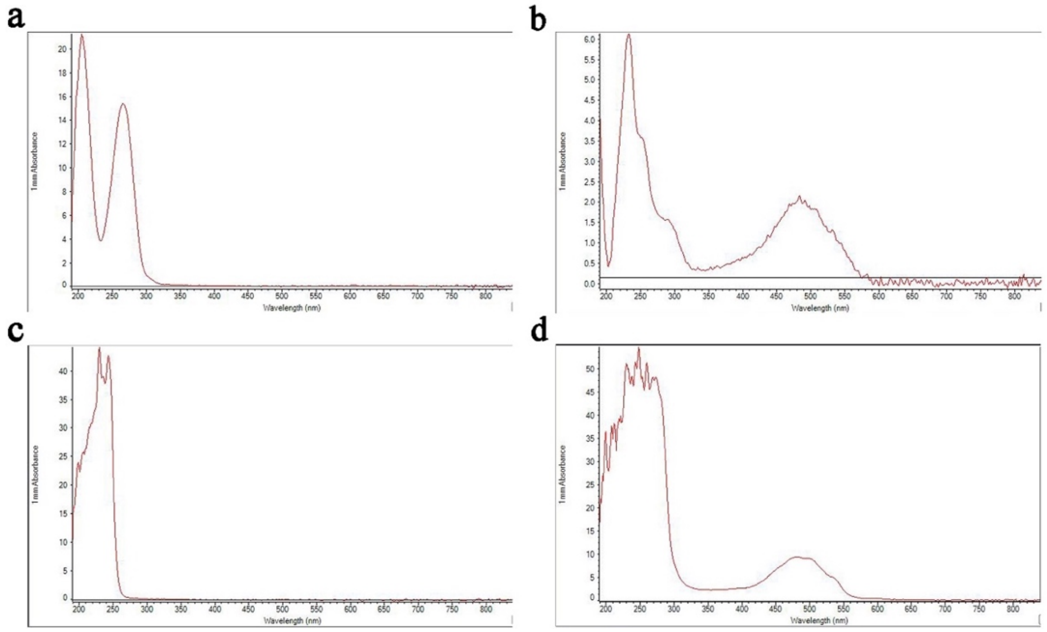

Figure S3. Ultraviolet spectra of (a) 5FU; (b) DOX; (c) PLGA; and (d) PLGA-5FU-DOX.

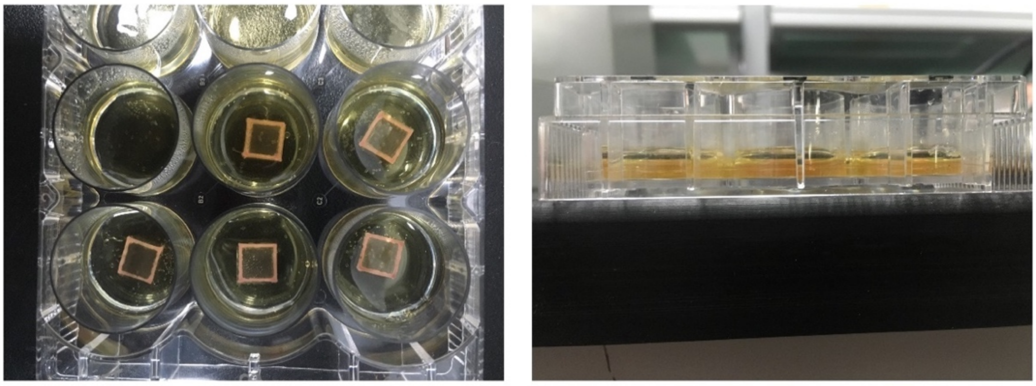

Figure S4. Preparation of sandwich-structured intelligent scaffolds.

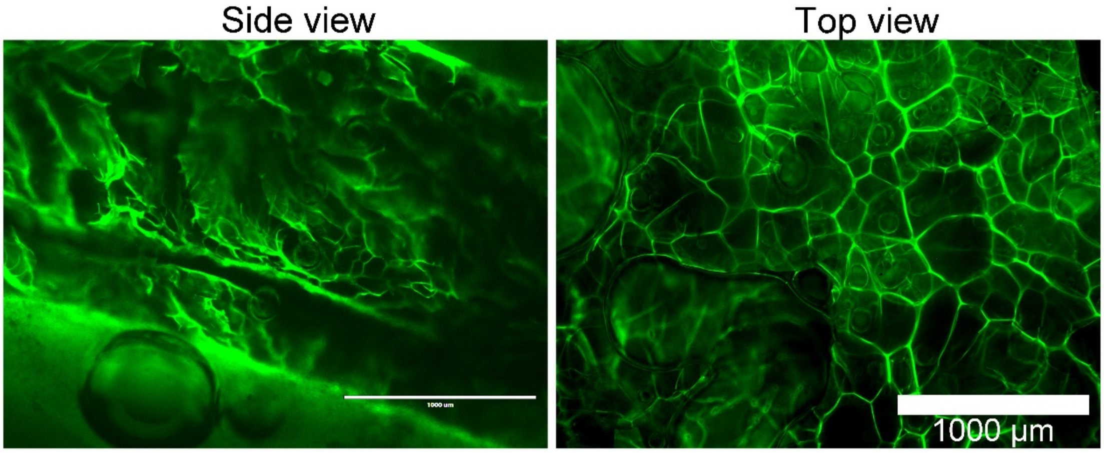

Figure S5. Fluorescence images of scaffolds.

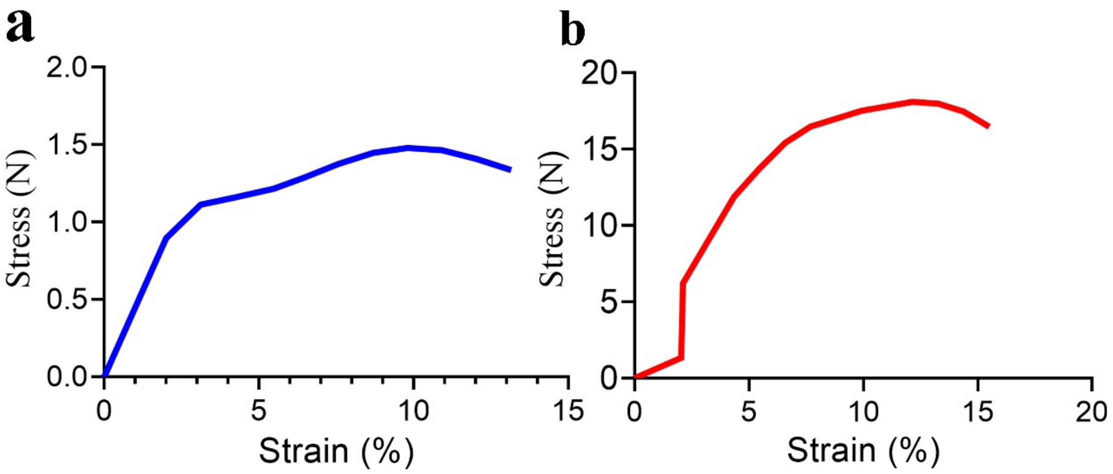

Figure S6. Tensile mechanical properties of GC scaffold (a) and IS (b).

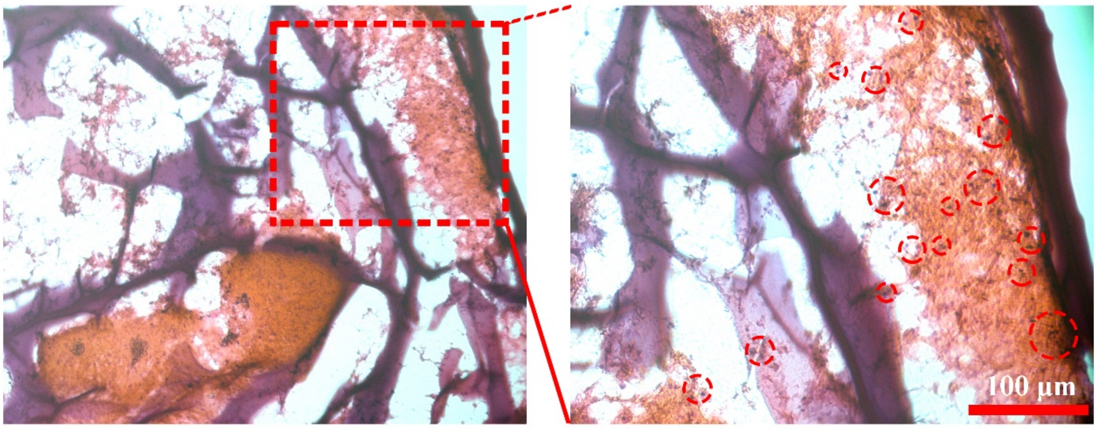

Figure S7. H&E staining of scaffolds after dropping blood for 5 minutes (the red circles indicate infiltrated cells).

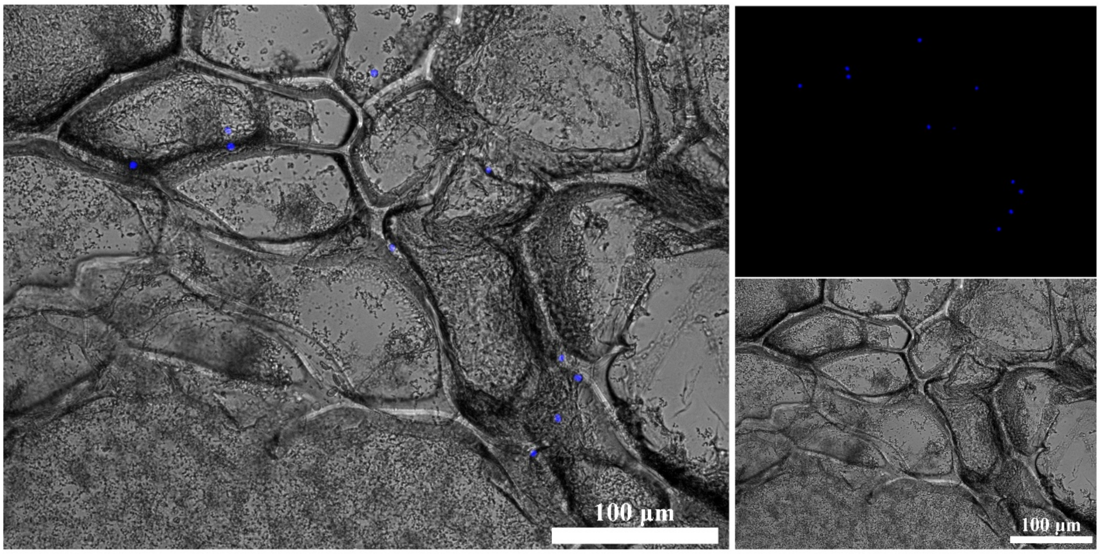

Figure S8. Bright field and fluorescent images of scaffold after dropping blood for 5 minutes (blue: DAPI).

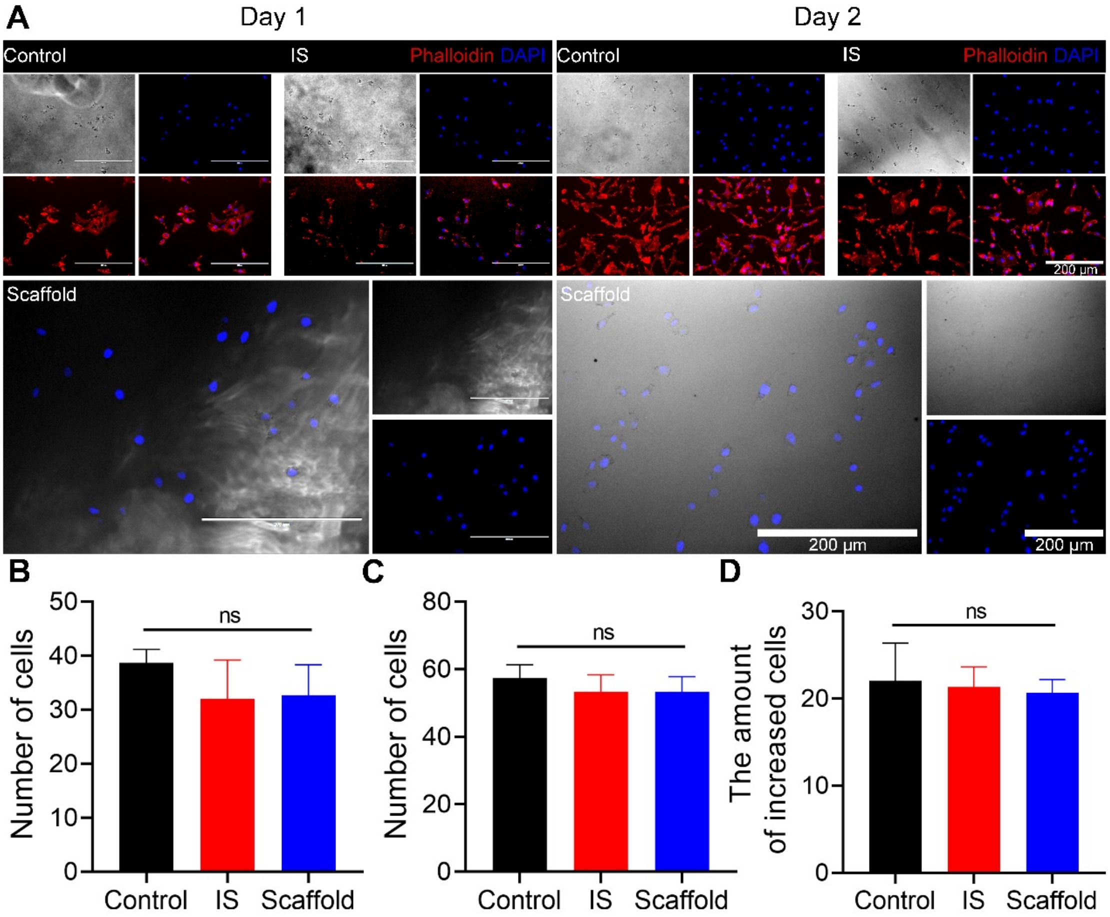

Figure S9. Assessment of biocompatibility of the scaffolds using MDA-MB-231 cells (untreated cells were used as the control, IS indicates cells on the culture dishes

following the addition of IS, and Scaffold indicates cells on IS scaffolds). (A) Immunofluorescence images on days 1 and 2. (B) The number of surviving cells on day 1. (C) The number of surviving cells on day 2. (D) Compared with the first day, the number of cells increased on the second day.

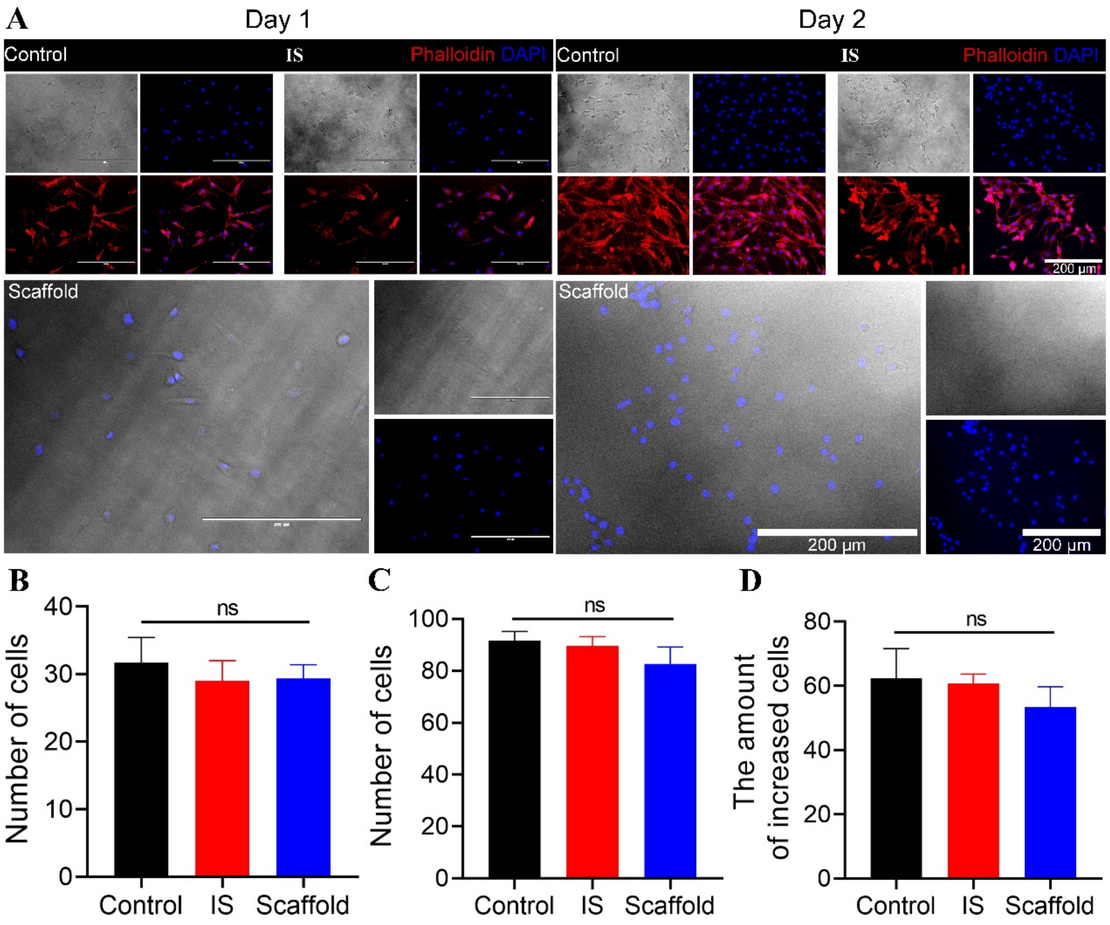

Figure S10. Assessment of biocompatibility of scaffolds using NIH3T3 cells. (A) Immunofluorescence images on days 1 and 2. (B) The number of surviving cells on day 1. (C) The number of surviving cells on day 2. (D) Compared with the first day, the number of cells increased on the second day. Control, untreated cells; IS, cells on culture following the addition of IS; Scaffold, cells on IS scaffolds.

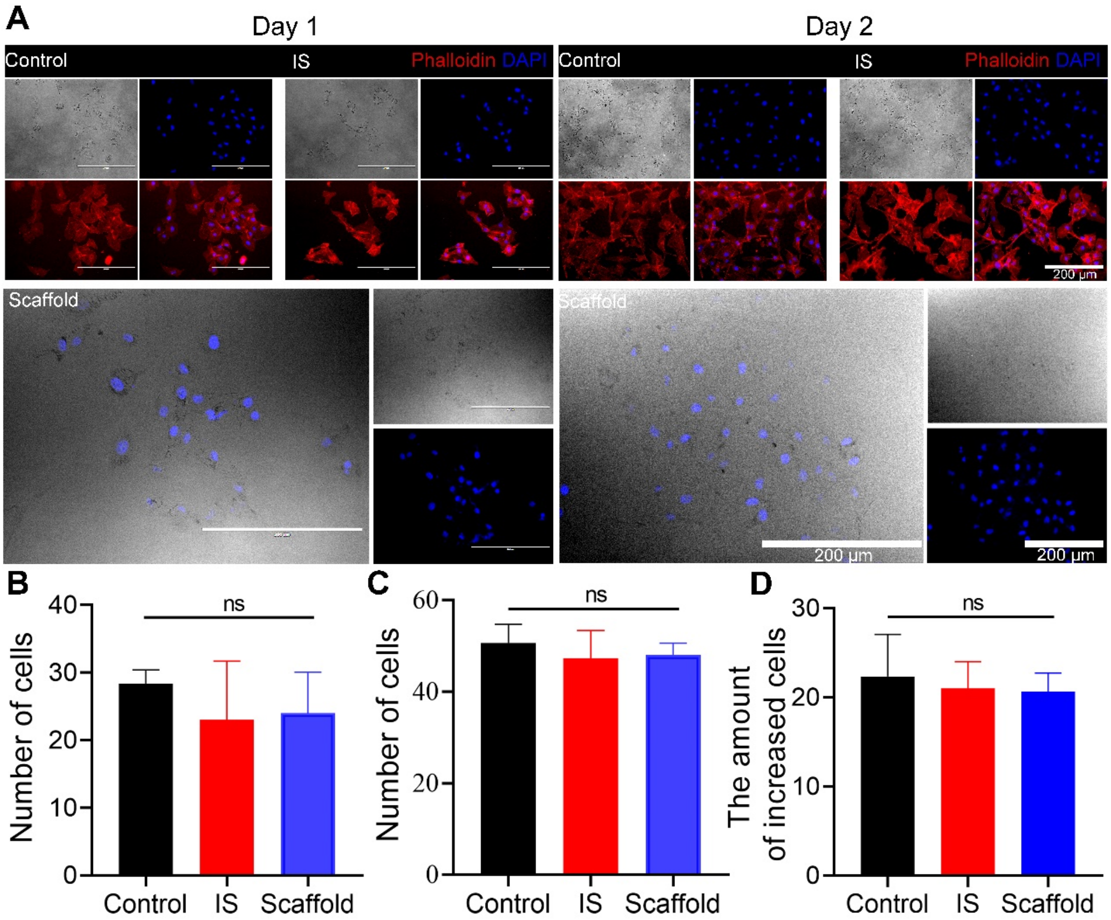

Figure S11. Assessment of biocompatibility of the scaffolds using HUVEC cells. (A) Immunofluorescence images on days 1 and 2. (B) The number of surviving cells on day 1. (C) The number of surviving cells on day 2. (D) Compared with the first day, the number of cells increased on the second day. Control, untreated cells; IS, cells on culture following the addition of IS; Scaffold, cells on IS scaffolds.

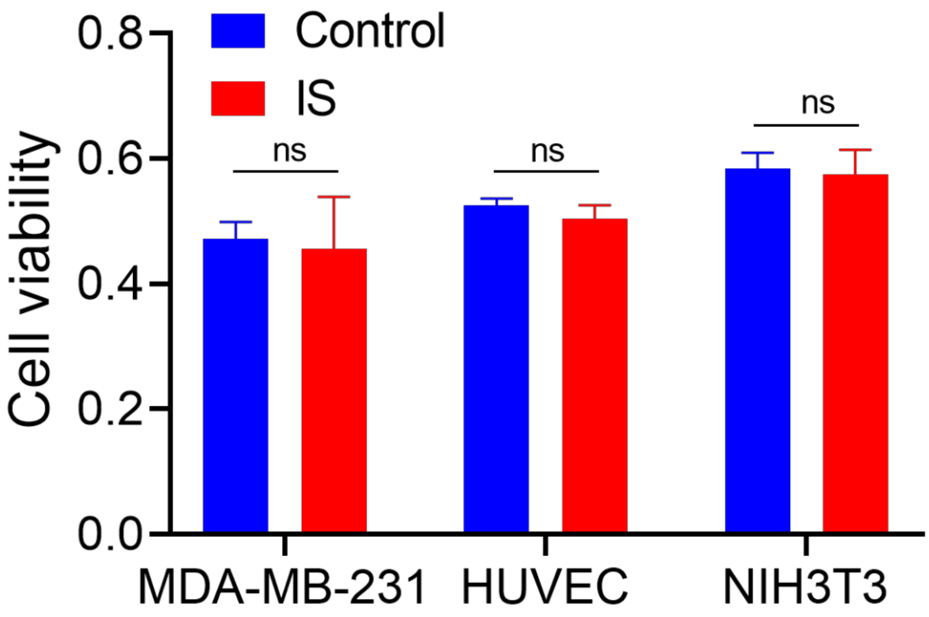

Figure S12. Cell viability after incubation with IS for 2 days.

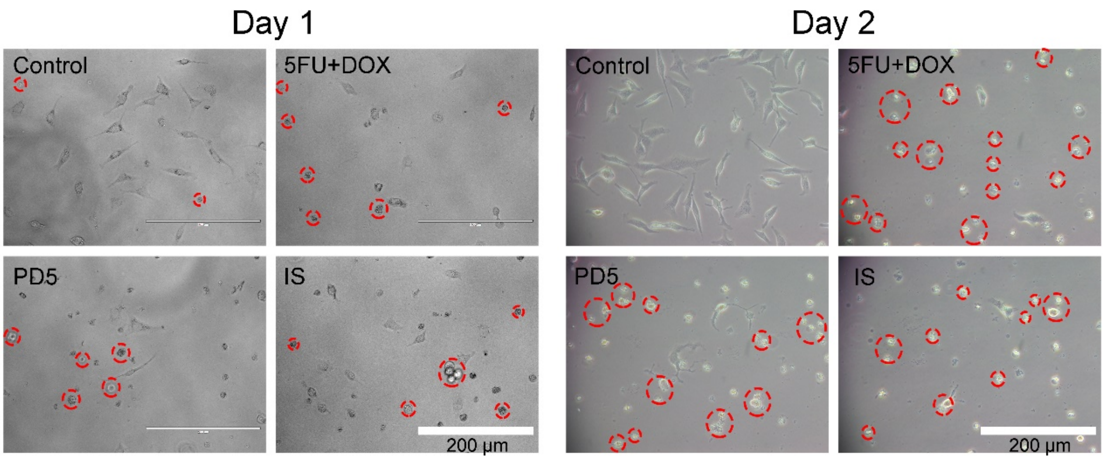

Figure S13. Bright field images of cells treated with DOX+5FU mixed solution, PD5 scaffolds, and IS, respectively; untreated cells were used as the control (the red circles indicate dead cells).

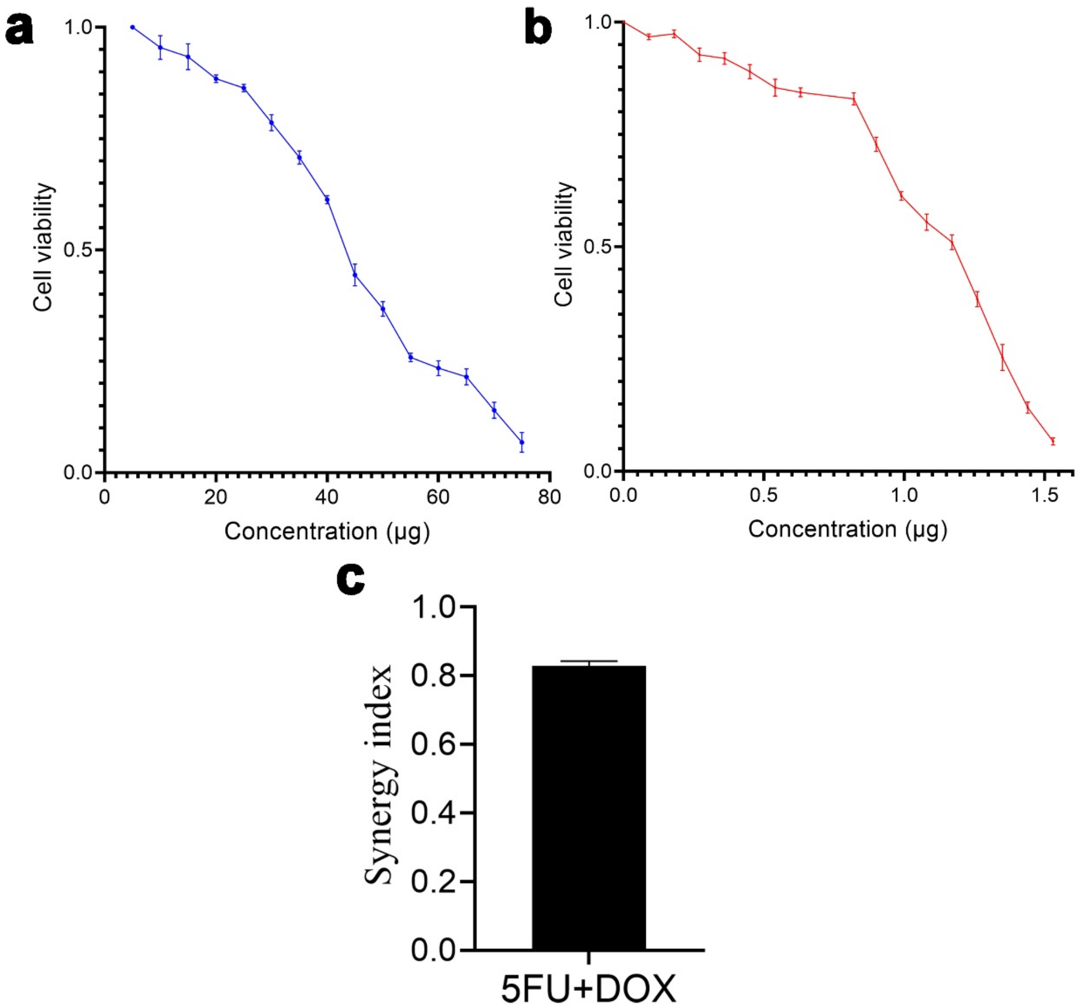

Figure S14. Cell survival after treatment. (a) Cell viability of MDA-MB-231 cells treated with different concentrations of 5FU; (b) cell viability of MDA-MB-231 cells treated with different concentrations of DOX; (c) synergistic index at the concentration of 0.544 μg/mL DOX and 2 μg/mL 5FU.

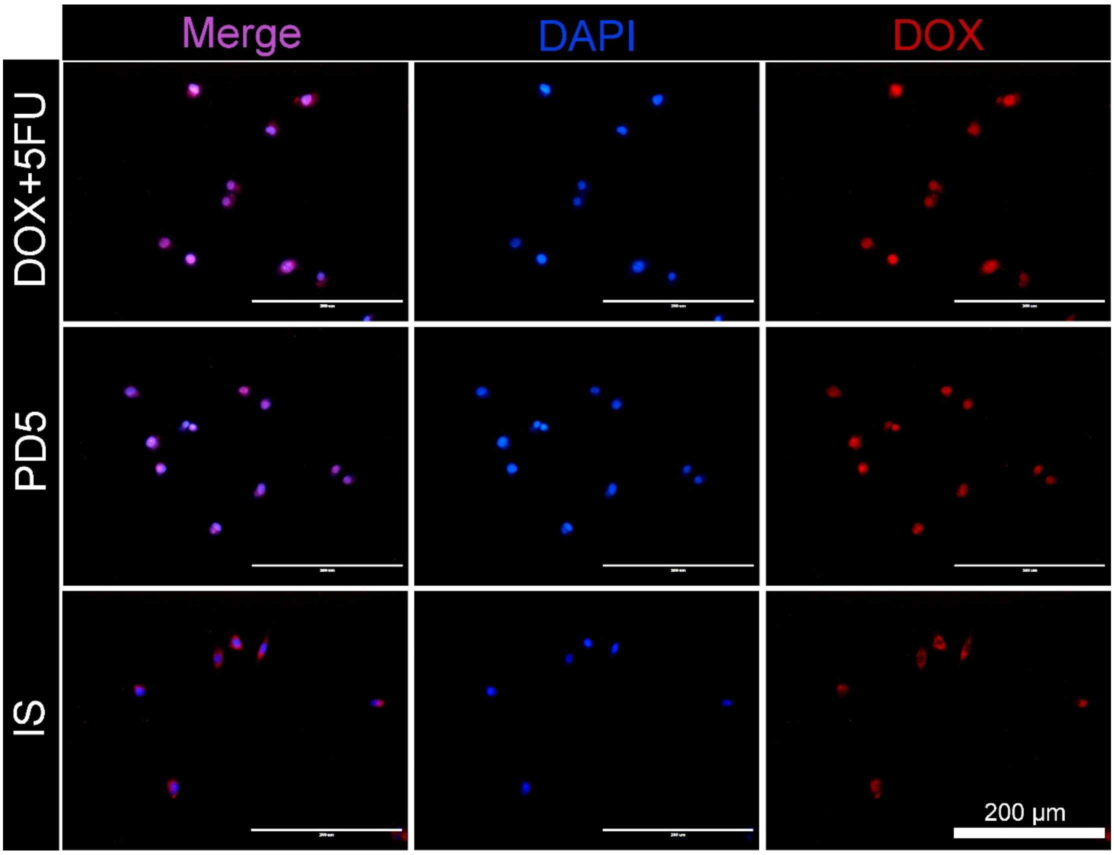

Figure S15. The drug uptake in cells after treatment for 8 h.

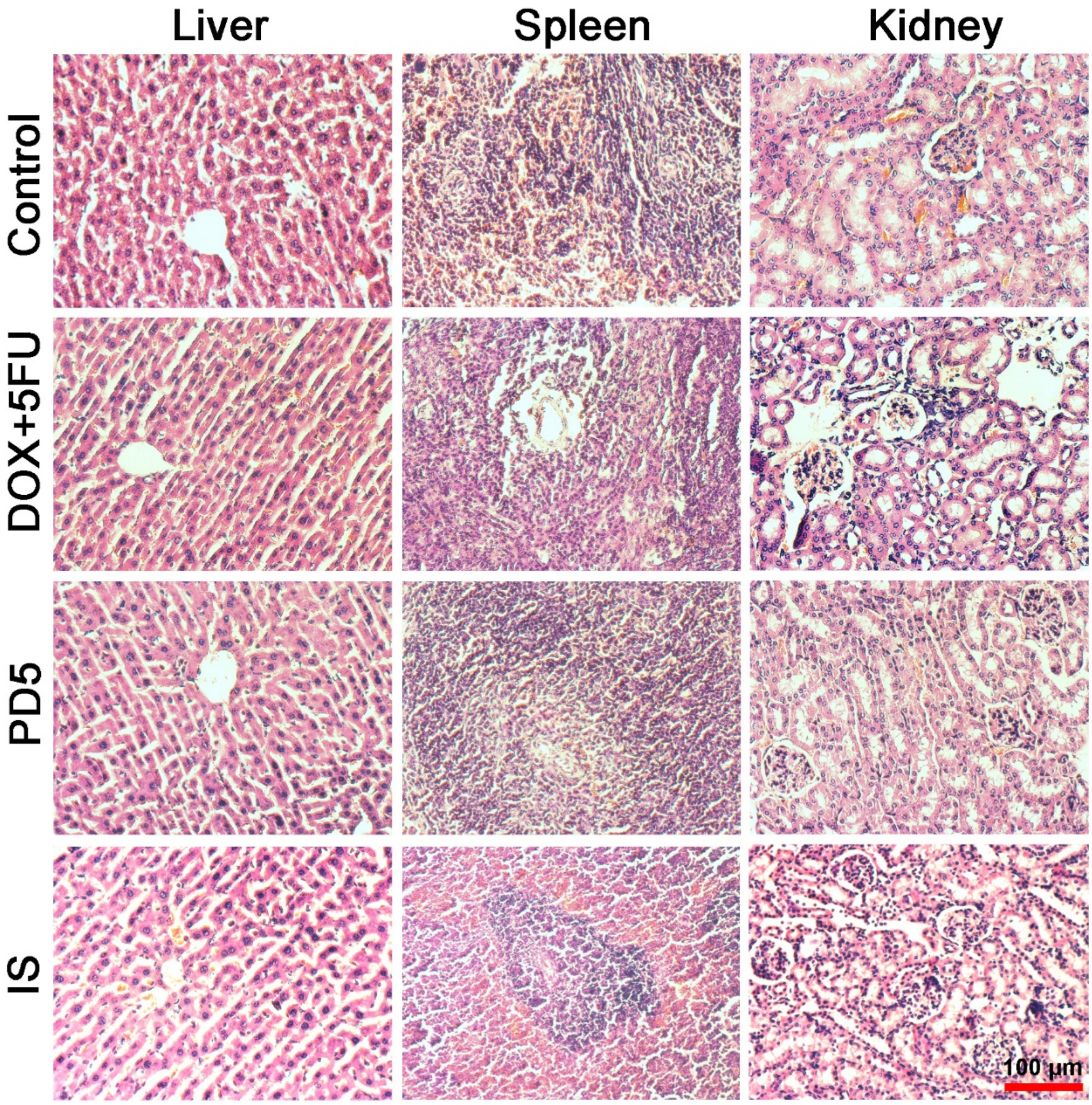

Figure S16. H&E Staining of spleen, lung, and kidney of mice in the different treatment groups.

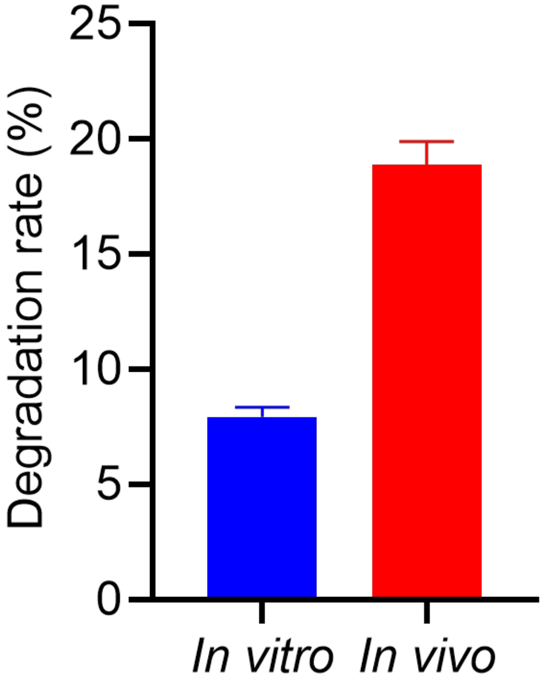

Figure S17. Degradation rate of IS after 30 days.
